# The impact of fibronectin knockout on invasion and migration of endometrial cell in adenomyosis

**DOI:** 10.1016/j.heliyon.2023.e19674

**Published:** 2023-08-30

**Authors:** Jiangman Gao, Wei Guo, Rong Li, Jie Qiao, Xiaoyu Long

**Affiliations:** a State Key Laboratory of Female Fertility Promotion, Center for Reproductive Medicine, Department of Obstetrics and Gynecology, Peking University Third Hospital, Beijing, 100191, China; b National Clinical Research Center for Obstetrics and Gynecology (Peking University Third Hospital), Beijing, 100191, China; c Key Laboratory of Assisted Reproduction (Peking University), Ministry of Education, Beijing, 100191, China; d Beijing Key Laboratory of Reproductive Endocrinology and Assisted Reproductive Technology (Peking University Third Hospital), Beijing, 100191, China

**Keywords:** Adenomyosis, Fibronectin, Endometrial cell, Invasion, Migration

## Abstract

The present study aimed to investigate the potential effect of fibronectin (FN) in adenomyosis progression. Small guide RNAs were designed to knock down FN expression in Ishikawa cells. The impact of FN on the proliferation, apoptosis, migration, and invasion of the cells was assessed. Cell proliferation was detected using a Celigo Imaging Cytometer system; apoptosis was quantified by flow cytometry; and cell migration and invasion were investigated via transwell assays. Cell proliferation was markedly suppressed in the FN knockout (KO) group compared with the control group, while apoptosis significantly increased. The levels of cell migration and invasion in the KO group were significantly decreased compared with the control group. Our study revealed that downregulation of FN expression is likely to restrain cell proliferation, migration, and invasion in endometrial cells in adenomyosis.

## 1 Introduction

Adenomyosis is a common gynaecological disorder in women of reproductive age and seriously impacts the health and well-being of women. The clinical symptoms include abnormal uterine bleeding, pelvic pain, dyspareunia, infertility, and adverse obstetric outcomes. This condition is characterized by the presence of endometrial glands and stroma within the myometrium, where they cause hyperplasia and hypertrophy of surrounding smooth muscle cells [1]. However, the pathogenic mechanisms of adenomyosis remain unclear. Elucidating the pathogenesis of adenomyosis will contribute to the development of targeted therapies to alleviate the symptoms of adenomyosis.

Enhanced direct invagination of the endometrial basalis into the inner myometrial layer might be a key feature leading to adenomyosis [2,3]. The endometrium can slide through bundles of weak smooth muscle fibres with loosened tissue cohesion. Fibrosis is also a pathological feature of adenomyosis [4]. Activated myofibroblasts might be critical effector cells in adenomyosis by secreting fibrogenic mediators [5]. Adenomyosis-related fibrosis is characterized by progressive accumulation of extracellular matrix (ECM) components, including collagen, fibronectin (FN) and alpha smooth muscle actin (αSMA) [4]. In the development of adenomyosis, dysregulation of ECM function may facilitate endometrial cell migration and invasion into the myometrium [2] and may also contribute to adenomyosis-related fibrosis [4].

FN is a ubiquitous glycoprotein in both blood plasma and the ECM. FN, as one of the most abundant components of the ECM, has diverse biological roles in development, cellular growth and differentiation, adhesion, migration, and wound healing, mainly through integrin-mediated signalling [[6], [7], [8], [9]]. Plasma FN is synthesized by hepatocytes and secreted into the blood plasma in a soluble, compact, inactive form [10,11]. Some diseases, such as inflammation, ischaemic heart, and stroke, cause vascular tissue damage, increasing blood plasma FN [[12], [13], [14]]. Cellular FN is a mixture of isoforms and is synthesized by many kinds of cells, including fibroblasts, endothelial cells, chondrocytes, synovial cells, and myocytes [15]. In the ECM, the monomeric FN molecule interacts with nearby FN molecules and other ECM proteins to form large, polymeric structures that support cell growth, wound healing, and tissue repair [10,11,16]. FN has been proposed to play an essential role in the pathobiology of cancer, such as in cell proliferation, migration, invasion, and metastasis [11,[17], [18], [19]], although its function in tumorigenesis and malignant progression is highly controversial [20]. Our previous study demonstrated that serum FN was significantly upregulated in women with adenomyosis [21].

The role of FN in the pathogenesis of adenomyosis and its potential predictive value for diagnosis or prognosis remain unclear. In this study, we used the Ishikawa cell line to evaluate the role of FN in cell proliferation, apoptosis, migration, and invasion of the endometrium to elucidate the potential function of FN in the pathogenesis of adenomyosis.

## 2 Materials and methods

### 2.1 Cell culture

This study was approved by the Institutional Review Board of Peking University Third Hospital. The Ishikawa cell line, a well-differentiated endometrial adenocarcinoma cell line, was purchased from Sigma-Aldrich (Shanghai, China). A frozen vial of Ishikawa cells was completely thawed in a 37 °C water bath. The cells were placed in a centrifuge vial for 3 min at 1300 rpm. The outside of the cryotube was disinfected with 75% ethanol, and the tube was transferred to a biosafety hood. The freezing medium was discarded, and the cells were resuspended in 1 ml of warm supplemented medium, Dulbecco's modified Eagle's medium (DMEM, Corning) containing 10% foetal bovine serum (FBS, Ausbian, cat. no. VS500T). Cells were seeded in a 6-cm dish containing 3 ml of supplemented medium and incubated in a humidified atmosphere containing 5% CO_2_ at 37 °C. The temperature regime and atmospheric pressure were controlled during incubation of isolated cells according to previous studies [22,23]. The culture medium was changed 24 h after seeding and every other day thereafter until the culture reached 80% confluency.

### 2.2 Cell transfection

Cells in the logarithmic growth phase were digested with trypsin. Cell suspensions (1–2 × 10^5^ cells/ml) were prepared with supplemented medium and seeded in 6-well plates to achieve a 15–30% area of the Petri dish after 24 h. For knockout of endogenous FN expression, the plasmids (FN1-sgRNA) (6 × 10^8^ TU/ml) were cotransfected at the indicated amounts into 2 × 10^5^ cells as the knockout (KO) group. The CON-sgRNA plasmids (5 × 10^8^ TU/ml) were transfected as the negative control of the knockout (NC–KO) group. After 8–16 h of transfection, we observed the cell state and replaced the transfection medium with conventional medium. The criteria of successful transfection for the subsequent experiment were as follows: 1) cells were in good condition without mass death and were at a similar state in the NC-KO group and the KO group; 2) the transfection efficiency was higher than 70%.

### 2.3 Cell number analysis

The cells in the logarithmic growth phase were digested, resuspended, and counted to ensure that the density was 1500 cells per well after seeding. Cell suspensions (100 μl) were added to wells and cultured in an incubator at 37 °C and 5% CO_2_. From the second day, the cells were counted using the Celigo® adherent cell cytometry system (Nexcelom, Lawrence, MA, USA) according to methods described previously [24] once a day for five consecutive days. The cell growth curve was drawn according to cell count and time points. The fold change in proliferation was the ratio of the cell count at each time point to the cell count on day 1 of the group.

### 2.4 Flow cytometry

Cells were digested, resuspended, and assessed by flow cytometry based on cell binding to Annexin V. Three wells were set for each group, and the number of cells in each well was more than 5 × 10^5^. Cell precipitation was obtained by centrifugation at 1300 rpm for 15 min, and the cells were washed twice with PBS. The precipitated cells were washed with 1 × binding buffer once and centrifuged at 1300 rpm for 3 min. After centrifugation, the cells were resuspended in 200 μl of 1 × binding buffer, and 10 μl of Annexin V-APC (eBioscience, cat. no. 88–8007) was added to the cells. After incubation for 15 min at room temperature in the dark, 800 μl of 1 × binding buffer was added, and the cells were analyzed by flow cytometry (BD Biosciences, cat. no. C6 PLUS, USA).

### 2.5 Transwell assays for cell migration

The migratory capacity of Ishikawa cells was determined using a 24-well Transwell chamber with 12 pores (Corning, cat. no. 3422, USA). The Transwell kit was prepared, and the required number of chambers was placed in a 24-well plate. Then, 100 μl of serum-free medium was added to the upper chamber and incubated at 37 °C for 1 h. Cells were resuspended in serum-free medium and counted. The medium was removed carefully, 100 μl of cell suspension (1 × 10^5^ cells/well) was seeded in the upper chamber, and 600 μl of culture medium with 30% FBS was added to the lower chamber. Transwell chambers were incubated at 5% CO_2_ and 37 °C for 24 h. Nonmigrated cells on the filter side of the upper chamber were removed with a cotton swab. The cells were fixed with 4% paraformaldehyde for 30 min and stained with Giemsa solution (Sigma, cat. no. 32884). The staining solution was dropped on the lower surface of the chamber to stain transfected cells for 3–5 min. The chambers were washed with 1 × PBS several times and air dried. The field of view was randomly selected, 5 photos at 200 × were taken from each chamber, and the migrated cells were counted.

### 2.6 Transwell assays for cell invasion

The invasive capacity of Ishikawa cells was determined by using a Transwell chamber covered with Matrigel. The invasion kit was placed in a biosafety hood until it melted at room temperature. The chambers were placed in a new 24-well plate. The upper and lower chambers were loaded with 500 μl of serum-free medium and incubated in an atmosphere of 5% CO_2_ at 37 °C for 2 h for Matrigel rehydration. The cells were digested and resuspended in serum-free medium. The number of cells was adjusted according to the pre-experiment, usually 1 × 10^5^ cells/well (24-well plate). After complete Matrigel rehydration, the chambers were transferred into a new 24-well plate. The medium in the upper chamber was removed, and 200 μl of cell suspension was added. The lower chamber was loaded with 750 μl of 30% FBS medium and cultured in an incubator at 5% CO_2_ and 37 °C for 24 h. Noninvasive cells in the upper chamber were removed with a cotton swab. The cells were fixed with 4% paraformaldehyde, stained with Giemsa solution and imaged under a microscope (Olympus, Tokyo, Japan). Microscopy was used to count the number of invasive cells in five random visual fields (200 × photos). The invasive ability of the cells is indicated by the number of migratory cells.

### 2.7 Statistical analysis

Three samples were included in each group, and three replicates were used in each experiment. All data are presented as the mean ± standard deviation (SD) and were evaluated using SPSS 23.0 software (IBM). The statistical significance of differences between the groups was determined using two-tailed *t*-test. A *P* value < 0.05 was defined as statistically significant.

## 3 Results

### 3.1 Cell proliferation and apoptosis

**Fig. 1:**
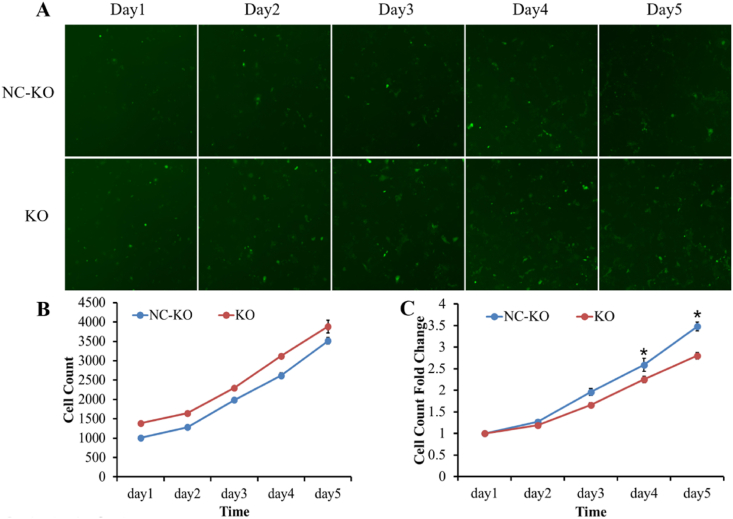
The proliferation of cells in the control and infection groups. (A) Images of cells on 5 consecutive days after infection (100 × ); (B) Cell count assessed by the Celigo Cell Counting assay; (C) The proliferation rates of Ishikawa cells. *P < 0.05 vs. the negative control group.

The Celigo cell counting system was used to visualize and calculate the proliferative rate of transfected Ishikawa cells. The proliferation of cells in the NC-KO group and KO group is shown in Fig. 1. Fig. 1A shows the images of cells on 5 consecutive days after infection, Fig. 1B shows the cell counts every day, and Fig. 1C shows the cell proliferation rates compared to the cell counts on day 1. On day 4 and day 5 post-transfection, the proliferation rate of cells in the KO group was markedly inhibited compared with that of cells in the NC-KO group (2.25 ± 0.08 vs. 2.59 ± 0.15, *P* < 0.05 and 2.80 ± 0.08 vs. 3.48 ± 0.10, *P* < 0.05), as shown in Fig. 1C and Supplemental Table 1.

**Fig. 2:**
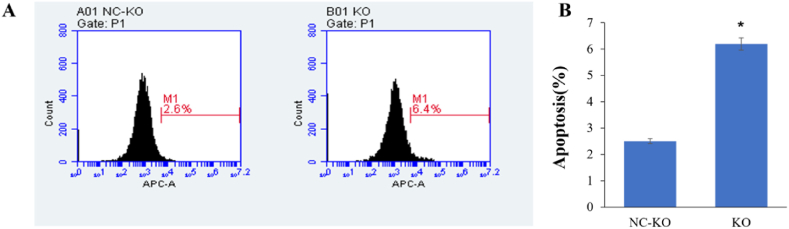
Analysis of cell apoptosis. Three replicates were set for each group. (A) The number of APC-A-positive cells detected by flow cytometry in the FN KO and NC-KO groups; (B) The histogram shows the percentage of apoptotic cells between the KO and NC-KO groups. *P < 0.05 vs. the NC-KO group.

Flow cytometry was performed to detect cell apoptosis. Fig. 2 shows analysis of cell apoptosis. Fig. 2A shows the number of APC-A-positive cells detected by flow cytometry, and Fig. 2B shows the percentage number of apoptotic cells in the KO and NC-KO groups. Compared with that of the NC-KO group, cell apoptosis was significantly increased in the KO group (6.19 ± 0.23 vs. 2.50 ± 0.10, *P* < 0.05) (Fig. 2C).

### 3.2 Cell migration and invasion

**Fig. 3:**
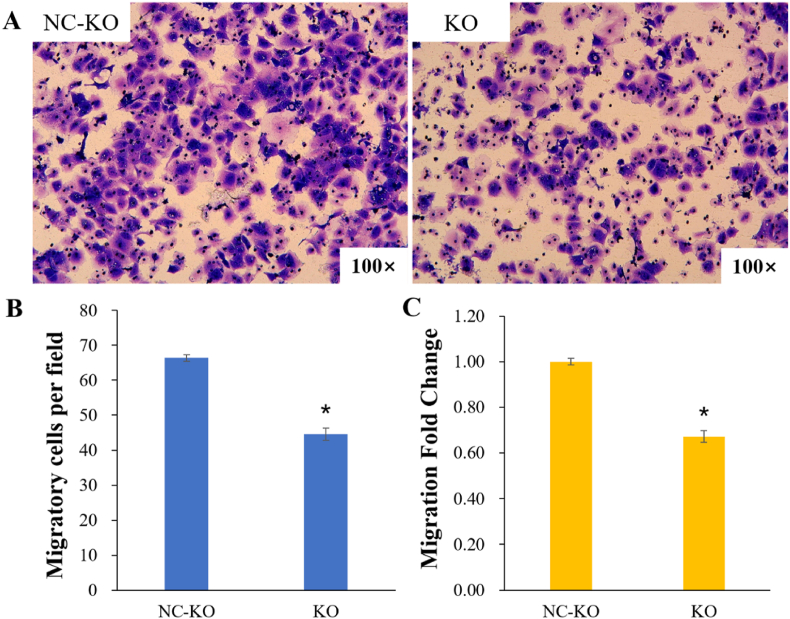
Transwell assays for cell migration. (A) Images of migrating cells; (B) The number of cells per field migrating to the Transwell compartment; (C) Migration fold change in the KO group and the NC-KO group. *P < 0.05 vs. the NC-KO group.

**Fig. 4:**
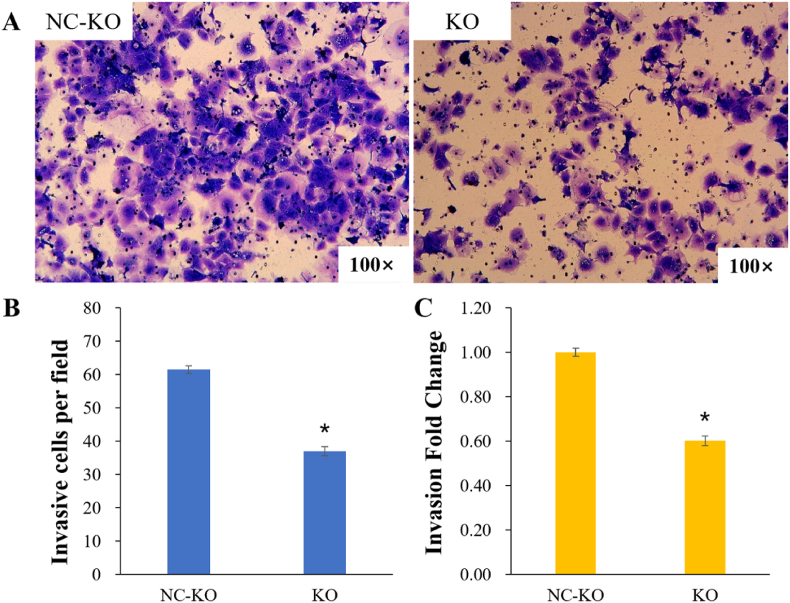
Transwell assays for cell invasion. (A) Images of invading cells; (B) The number of cells per field invading the Transwell compartment; (C) Invasion fold change in the KO group and the NC-KO group. *P < 0.05 vs. the NC-KO group.

Transwell assays were employed to measure cell migration and invasion after transfection, and the results were showed in Fig. 3, Fig. 4. Fig. 3A shows the images of migrating cells, and Fig. 4A shows the images of invading cells. Histograms show the cell counts per field migrating or invading to the transwell compartment in the KO and NC-KO groups and the fold change of cell counts compared to the NC-KO group. Tabular data is shown in Supplemental Table 2. Compared to that of the NC-KO group, the cell migration was significantly reduced (migratory cells per field: 45 ± 1.69 vs. 66 ± 0.94, *P* < 0.05; migration fold change: 0.67 ± 0.03 vs. 1.00 ± 0.01, *P* < 0.05) [Fig. 3 (B, C); Supplemental Table 2), and the cell invasion rate was substantially lower (invading cells per field: 37 ± 1.34 vs. 62 ± 1.14, *P* < 0.05; invasion fold change: 0.60 ± 0.02 vs. 1.00 ± 0.02, *P* < 0.05) in the KO group [Fig. 4 (B, C); Supplemental Table 2].

## 4 Discussion and conclusions

Adenomyosis is a gynaecological disorder resulting from pathological invasion of the endometrial basalis into the myometrium induced by enhanced cell survival, epithelial-to-mesenchymal transition (EMT), and cell migration. In addition, inflammation, tissue injury and repair have been proposed to be involved in the pathogenesis and pathophysiology of adenomyosis [2]. Given the role of FN in the migration and invasion of cancer cells and wound healing [20,25], as well as the upregulated serum FN in women with adenomyosis [21], we explored the potential function of FN in the pathological mechanism of adenomyosis. We found that in Ishikawa endometrial epithelial cells, downregulated FN expression increased cell apoptosis and decreased cell proliferation, migration and invasion.

The endometrial tissue in women with adenomyosis displays fewer apoptotic bodies and more rapid proliferation than that in healthy women [26]. Enhanced proliferation and survival of endometrial cells, along with enhanced migratory properties, may enable these cells to invade the endometrial myometrial interface [27]. FN is a relatively large molecule that can bind to multiple cell surface receptors, stimulating cell proliferation, supporting survival and promoting differentiation. In keloid fibroblasts, knockdown of FN suppressed cell proliferation and collagen deposition via the AKT/ERK signalling pathway [28]. Ji et al. found that FN suppressed the apoptosis of human trophoblasts by activating the PI3K/Akt signalling pathway [29]. Li et al. showed that downregulation of FN induced apoptosis by increasing the Bax/Bcl-2 ratio [30]. Wu and his colleagues demonstrated that knockdown of FN induced mitochondria-dependent apoptosis in rat mesangial cells [31]. In this study, downregulated FN expression decreased cell proliferation and increased cell apoptosis in Ishikawa endometrial epithelial cells.

Increased endometrial cell migration and invasion facilitate implantation of ectopic endometrium into the myometrium [32]. EMT is a biological process by which epithelial cells lose their intercellular adhesions and acquire an invasive and metastatic phenotype. EMT has been proposed to contribute to the migration of endometrial epithelial cells into the myometrium in adenomyosis [33,34]. Metastatic cancer cells exhibit a stronger EMT phenotype with increased migration, invasion and proliferation [30]. EMT is characterized in part by enhanced expression of mesenchymal markers, such as FN, N-cadherin and vimentin [3]. Downregulation of FN increases the expression of E-cadherin and decreases the expression of N-cadherin and vimentin, which suggests that FN knockdown might suppress the EMT process. FN induces EMT in colorectal cancer cells and plays a pivotal role in promoting colorectal cancer metastasis [35]. FN expression promotes the proliferation, invasion, and migration of carcinoma cells [36,37]. Previous reports have demonstrated that downregulation of FN inhibits cell migration and invasion in colorectal cancer and neuroblastoma cells [38,39]. We found similar results: FN knockout prevented the migration and invasion of endometrial cells, probably by regulating the EMT process. However, decreased cell migration and invasion might be associated with reduced cell proliferation and increased apoptosis resulting from FN knockout.

In summary, we found that the suppression of FN resulted in reduced cell proliferation, increased apoptosis, and decreased migration and invasion in endometrial cells, suggesting that FN is involved in the pathology of adenomyosis. FN might play a pivotal role in endometrial cell invasion in patients with adenomyosis. However, in this study, we only observed changes at the cellular level, and further elucidation of the underlying mechanism and signalling pathways of FN that are associated with the pathogenesis of adenomyosis is needed to identify clinical therapeutic targets.

## Funding

This work was supported by the 10.13039/501100001809National Natural Science Foundation of China (81701407).

## Ethics statement

The study was approved by Peking University Third Hospital Medical Science Research Ethics Committee (IRB00006761-2015063).

## Author contribution statement

Jiangman Gao: Performed the experiments; Analyzed and interpreted the data; Wrote the paper. Wei Guo: Performed the experiments; Analyzed and interpreted the data. Jie Qiao and Rong Li: Conceived and designed the experiments; Contributed reagents, materials, analysis tools or data. Xiaoyu Long: Conceived and designed the experiments; Analyzed and interpreted the data; Contributed reagents, materials, analysis tools or data, Wrote the paper.

## Data availability statement

Data will be made available on request.

## Declaration of competing interest

The authors declare that they have no known competing financial interests or personal relationships that could have appeared to influence the work reported in this paper.
