# Supplementary material for: The impact of fibronectin knockout on invasion and migration of endometrial cell in adenomyosis
Source: Heliyon. 2023 Aug 30;9(9):e19674. doi: 10.1016/j.heliyon.2023.e19674 (PMC10558947; doi:10.1016/j.heliyon.2023.e19674)
Supplement: Multimedia component 1 [file mmc1.docx]

**Supplemental Table 1**

The proliferation of cells in two groups.

| Time | Cell count | | Cell count fold change | |
| --- | --- | --- | --- | --- |
|  | NC-KO | KO | NC-KO | KO |
| Day 1 | 1011±33 | 1387±41 | 1 | 1 |
| Day 2 | 1281±25 | 1644±44 | 1.27±0.05 | 1.19±0.02 |
| Day 3 | 1984±19 | 2296±49 | 1.96±0.08 | 1.66±0.05 |
| Day 4 | 2615±68 | 3121±17 | 2.59±0.15 | 2.25±0.08^*^ |
| Day 5 | 3515±81 | 3885±161 | 3.48±0.10 | 2.80±0.08^*^ |

**Note:** * *P*<0.05
