# Supplementary material for: The impact of fibronectin knockout on invasion and migration of endometrial cell in adenomyosis
Source: Heliyon. 2023 Aug 30;9(9):e19674. doi: 10.1016/j.heliyon.2023.e19674 (PMC10558947; doi:10.1016/j.heliyon.2023.e19674)
Supplement: Multimedia component 2 [file mmc2.docx]

**Supplemental Table 2**

Transwell assays for cell migration and invasion.

| Variable | NC-KO | KO |
| --- | --- | --- |
| Migratory cells per field | 66±0.94 | 45±1.69^*^ |
| Migration fold change | 1±0.01 | 0.67±0.03^*^ |
| Invasion cells per field | 62±1.14 | 37±1.34^*^ |
| Invasion fold change | 1±0.02 | 0.60±0.02^*^ |

**Note:** * *P*<0.05
